# The Contractile Apparatus Is Essential for the Integrity of the Blood-Brain Barrier After Experimental Subarachnoid Hemorrhage

**DOI:** 10.1007/s12975-018-0677-0

**Published:** 2018-11-23

**Authors:** Clara Luh, Sergej Feiler, Katrin Frauenknecht, Simon Meyer, Lubomir T. Lubomirov, Axel Neulen, Serge C. Thal

**Affiliations:** 1 Department of Anesthesiology, University Medical Center of the Johannes Gutenberg-University, Mainz, Germany; 2 Department of Neurosurgery, Inselspital, Bern University Hospital, Bern, Switzerland; 3 Institute of Neuropathology, University Hospital Zurich, University of Zurich, Zurich, Switzerland; 4 Institute of Vegetative Physiology, University of Cologne, Köln, Germany; 5 Department of Neurosurgery, University Medical Center of the Johannes Gutenberg-University, Mainz, Germany; 6 Center for Molecular Surgical Research (MFO), Medical Center of the Johannes Gutenberg-University, Langenbeckstrasse 1, 55131 Mainz, Germany

**Keywords:** Subarachnoid hemorrhage, Brain edema, ML-7, Myosin light chain kinases, Intracranial pressure, Blood-brain barrier

## Abstract

Development of vasogenic brain edema is a key event contributing to mortality after subarachnoid hemorrhage (SAH). The precise underlying mechanisms at the neurovascular level that lead to disruption of the blood-brain barrier (BBB) are still unknown. Activation of myosin light chain kinases (MLCK) may result in change of endothelial cell shape and opening of the intercellular gap with subsequent vascular leakage. Male C57Bl6 mice were subjected to endovascular perforation. Brain water content was determined by wet-dry ratio and BBB integrity by Evans-Blue extravasation. The specific MLCK inhibitor ML-7 was administered to the mice to determine the role of the contractile apparatus of the neurovascular unit in determining brain water content, BBB integrity, neurofunctional outcome, brain damage, and survival at 7 days after SAH. Inhibition of MLCK significantly reduced BBB permeability (Evans Blue extravasation − 28%) and significantly decreased edema formation in comparison with controls (− 2%). MLCK-treated mice showed reduced intracranial pressure (− 53%), improved neurological outcome at 24 h and 48 h after SAH, and reduced 7-day mortality. Tight junction proteins claudin-5 and zonula occludens-1 levels were not influenced by ML-7 at 24 h after insult. The effect of ML-7 on pMLC was confirmed in brain endothelial cell culture (bEnd.3 cells) subjected to 4-h oxygen-glucose deprivation. The present study indicates that MLCK contributes to blood-brain barrier dysfunction after SAH by a mechanism that does not involve modulation of tight junction protein levels, but via activation of the contractile apparatus of the endothelial cell skeleton. This underlying mechanism may be a promising target for the treatment of SAH.

**Electronic supplementary material:**

The online version of this article (10.1007/s12975-018-0677-0) contains supplementary material, which is available to authorized users.

## Introduction

A critical factor for the 35% mortality rate in patients suffering from subarachnoid hemorrhage (SAH) is the development of severe global cerebral edema [1]. In human studies, brain edema formation correlates with ischemic damage, cognitive deficits, and mortality [1, 2]. Post-hemorrhagic brain edema is mostly of vasogenic origin and results from the disruption of blood-brain-barrier (BBB) integrity [3]. The precise underlying mechanisms leading to BBB breakdown at the neurovascular level and the significance of the endothelial cells for the development of vasogenic brain edema after SAH are still unknown. Recent in vitro studies suggest that the activation of the contractile apparatus of endothelial cells may contribute to the disruption of the BBB in pathologic conditions [4–6]. Data from mechanical brain lesions indicate increased activity of myosin light chain kinase (MLCK) [7, 8] leading to the phosphorylation of regulatory myosin light chains (MLC). This finding was associated with the loss of BBB integrity and worsening of brain edema.

The aim of the present study was to determine the role of the cytoskeleton of vascular cells in BBB dysfunction, brain edema formation, intracranial pressure regulation, and neurological recovery after experimental SAH.

## Methods

### Experimental Subarachnoid Hemorrhage

All animal experiments were conducted in compliance with institutional guidelines of the Johannes Gutenberg-University, Mainz. The Animal Ethics Committee of the Landesuntersuchungsamt Rheinland-Pfalz approved all experiments. One hundred thirty-six male C57Bl6N mice (weight 18–24 g, age 12–14 weeks, Charles River Laboratory, Sulzfeld, Germany) had free access to food and water before and during the experiments. After induction of anesthesia by intraperitoneal injection of midazolam (5 mg/kg; Ratiopharm, Ulm, Germany), fentanyl (0.05 mg/kg; CuraMed, Karlsruhe, Germany), and medetomidin (0.5 mg/kg; Pfizer, Germany) animals were intubated and mechanically ventilated under control of the end-expiratory pCO_2_ levels with a mixture of 60% N_2_ and 40% O_2_ (Minivent, Hugo Sachs, Hugstetten, Germany).

SAH was induced by the endovascular puncture technique as previously described [9]. Rectal temperature was maintained at 37 °C with a thermostatically regulated, feedback-controlled heating pad (Hugo-Sachs). Intracranial pressure (ICP) was continuously recorded from 10 min before SAH until 15 min after SAH. ICP probe (Codman ICP microsensor, Johnson & Johnson Medical Limited, Berkshire, UK) was placed on the contralateral side 3 mm posterior of bregma. Regional cerebral blood flow (CBF) was determined in the territory of the middle cerebral artery (MCA) of the left hemisphere. A laser-Doppler flow (LDF) glass fiber probe was fixed to the skull, and LDF was determined with Periflux 4001 Master (Perimed, Stockholm, Sweden) and recorded continuously (5-Hz sampling rate) from 10 min before SAH until 15 min after SAH.

For induction of SAH, animals were placed in the supine position and the neck was incised along the midline. After preparation of the left bifurcation of the external (ECA) and internal carotid arteries (ICA), a 5-0 monofilament (Prolene, Ethicon, Johnson & Johnson Medical Limited, Berkshire, UK) was advanced via the ECA into the ICA until a slight decrease of the ipsilateral CBF was detected as indicated by LDF. This ensured that the position of the tip of the filament was close to the bifurcation of the ICA, thus reducing the flow to the MCA. The filament was advanced 1 mm further until the ICA was perforated near its intracranial bifurcation. Subsequently, the filament was withdrawn into the external carotid artery resulting in reperfusion of the ICA and SAH. The duration of the endovascular occlusion of the common carotid artery was at maximum 2–3 min.

At the end of the surgical preparation, wounds were closed with filament sutures and anesthesia was antagonized by intraperitoneal injection of atipamezol (2.5 mg/kg; Pfizer, Karlsruhe, Germany) and flumazenil (0.5 mg/kg; Hoffmann-La-Roche, Grenzach-Wyhlen, Germany). The animals usually awakened and regained normal motor function within 1–2 min. Afterwards, animals were placed in individual cages and recovered for 6 h in an incubator at 33 °C with 35% humidity (IC8000, Draeger, Germany) in order to prevent hypothermia [10].

### Oxygen-Glucose Deprivation (OGD)

Murine bEnd.3 cells (passage 23 to 30) were cultured in Dulbecco’s modified Eagle’s medium supplemented with 15% fetal calf serum (FCS), 100 IU/ml penicillin, and 100 μg/ml streptavidin. Cells were grown in an incubator at 37 °C with humidified (95%) air and 5% CO_2_. For experiments, 400,000 cells per well were seeded on poly-d-lysin coated 6-well plates. After 24-h medium was supplemented with 10 μM 1-(5-iodonapthalene-1-sulfonyl)-1H-hexahydro-1,4-diazepine hydrochloride (ML-7, Sigma-Aldrich, Germany) in dimethyl sulfoxide (DMSO) or DMSO as vehicle control (0.1% final concentration). After 30-min incubation, culture medium was removed, cells were washed with PBS, and serum- and glucose-free DMEM supplemented with DMSO or 10 μM ML-7 (0.1% final DMSO concentration). Hypoxic conditions (1% oxygen, 5% CO_2_) were established for 4 h in a hypoxic chamber. After 15 min of reoxygenation, supernatant was removed, and cells were washed with ice-cold PBS and lysed in 60 μl radioimmunoprecipitation assay (RIPA) buffer (50 mM Tris-HCl, pH 7.5; 150 mM NaCl; 1 mM EDTA; 1% NP-40; 0.1% sodium dodecyl sulfate [SDS], protease and phosphatase inhibitors [Roche]). Whole-cell lysates were centrifuged at 14,000*g* for 20 min and 4 °C, and supernatants were stored at − 80 °C until use.

### Experimental Protocol

Investigators were blinded to the treatment given to the animals. Mice were treated with intraperitoneal injection of the selective MLCK inhibitor ML-7 (1 mg/kg) or vehicle solution (0.9% NaCl) 1 h prior to and 6 h after SAH. ML-7 was stored as dry powder at − 20 °C and freshly dissolved in normal saline before use [7].

A. mRNA expression of MLCK, MLC, inflammatory cytokines, and tight junction proteins were determined in animals randomly assigned to three survival groups: SAH 12 and 24 h and sham-surgery (*n* = 8 each).
B. The effect of MLCK inhibition on ICP, neurological outcome, and hemispheric brain water content was determined 24 h after SAH in animals randomized to (1) naive vehicle, (2) SAH+vehicle, (3) naive+ML-7, and (4) SAH+ML-7 (*n* = 10 per group)
C. The effect of MLCK inhibition on BBB permeability after SAH was determined via Evans blue extravasation 24 h after SAH in animals randomized to (1) naive+vehicle, (2) SAH+vehicle, (3) naive+ML-7, and (4) SAH+ML-7 (*n* = 7 per group). In addition, the ICP was measured 24 h after SAH.
D. The effect of MLCK inhibition on physiological parameters, neurological outcome, and histological damage was determined 7 days after SAH via neuronal cell count in the hippocampus of animals randomized to (1) naive+vehicle, (2) SAH+vehicle, (3) naive+ML-7, and (4) SAH+ML-7 (*n* = 11 per group).
E. The influence of ML-7 on MLC phosphorylation in hypoxic brain endothelial cells (bEnd.3 cells) following 4-h brain-glucose deprivation (OGD): (1) OGD+vehicle and (2) OGD+ML7 (triplicates in two independent experiments).

### Measurement of Physiological Parameters

Systolic blood pressure was non-invasively (NIBP) monitored on the tail of the mice using a modified NIBP system (RTBP 2000, Kent, USA) as previously described [10]. Cuff pressure signals were recorded with a sample rate of 100 Hz (A/D converter: PCI 9112, Adlink Technology, Taiwan; PC software: Dasylab 5.0, measX, Germany) and analyzed offline (Flexpro 6.0, Weisang, Germany) for systolic blood pressure and heart rate. In all animals, NIBP was measured 5 min before and 5 min after SAH. In mice surviving 7 days NIBP were measured daily.

In animals surviving 24 h, ICP values were determined and blood samples were drawn from the carotid artery. Arterial blood gas concentration, blood glucose levels, hematocrit, and electrolytes were determined with a blood gas analyzer (ABL800, Radiometer Medical, Denmark).

### Measurement of Brain Water Content

Twenty-four hours after SAH, mice were anesthetized by intraperitoneal anesthesia (midazolam, fentanyl, medetomidine). After removal, brains were sectioned along the midsagittal plane and the cerebellum removed. Each hemisphere was weighed and then dried for 24 h at 110 °C to determine the dry weight. The water content was assessed based on gravimetrical differences [11].

### Histological Evaluation

Seven days after SAH, mice were sedated by intraperitoneal anesthesia (midazolam, fentanyl, medetomidine) and exsanguinated by transcardiac perfusion using 2% paraformaldehyde (PFA) solution. The brains were carefully removed, post-fixed in 2% PFA, and embedded in paraffin. Coronal sections (4 μm) were cut at the level of the bregma (1.28, Mouse Brain Library Atlas; www.mbl.org) in order to visualize the hippocampus and subsequently stained with cresyl violet. An investigator blinded to the experimental groups determined the neuronal cell count (field 0.15 × 0.30 mm) in the CA1, CA2, and CA3 regions of the hippocampus.

### Extravasation of Evans Blue

A previously reported quantitative assay was used to determine Evans Blue levels in the ipsilateral hemisphere [7]. Three hours after induction of SAH or in naive animals, 1 ml of 0.4% Evans Blue (Sigma-Aldrich, Munich, Germany) was injected intraperitoneally. Twenty-four hours after SAH, animals were exsanguinated by transcardiac perfusion. A fluorescent plate reader (MRX II, Dynex Technologies, Berlin, Germany) was used to measure intracerebral Evans Blue dye concentrations at 630 nm. The amount of extravasated Evans Blue was expressed as optical density (OD) per gram of hemispheric brain tissue.

### Quantification of Functional Outcome

Functional outcome before and after SAH was determined by an investigator blinded to the experimental groups. Body weight and a neuroscore were assessed. The 31-point neuroscore, which consists of tasks evaluating motor ability, alertness, balancing, and general behavior, was modified on the basis of an established protocol for rats [12] and the neurological scale of Bederson [13]. For each failed task, the mouse was assigned 1 point. Healthy mice were successful in all tasks and received a score of 0 (Supplemental Table S1). Body weight was determined daily and compared to values prior to SAH.

### Gene Expression Analysis

The mRNA quantification by real-time quantitative polymerase chain reaction (qPCR) was performed as previously described [14]. In brief, RNA was isolated from hippocampus and cortex samples using Qiagen-RNeasy plus universal kit (Qiagen) and reverse-transcribed into cDNA with QuantiTect reverse transcription Kit (Qiagen) according to the manufacturer’s instructions. cDNA of each sample was amplified by real-time Lightcycler 480 PCR System (Roche). RT-PCR for the detection of cyclophilin A (PPIA), myosin light chain kinase (MLCK) and myosin light chain (MLC), interleukin 1β (IL-1β), tumor necrosis factor α (TNFα), interleukin 6 (IL-6), zonula occludens-1 (ZO-1), Claudin 5 (Cl5), and occludin (Ocln) was carried out as previously reported using the real-time Lightcycler 480 PCR system (Roche) [7]. Equal amounts of cDNA (1 μl) were used in duplicates and amplified with Roche Lightcycler® 480 Probes Master (PPIA, IL-1β, Ocln), 480 SYBR Green I Master (TNFα, IL-6) or Thermo Scientific Absolute Blue SYBR Green (MLCK, MLC, ZO-1, Cl5). The absolute copy numbers of the target genes were normalized against the absolute copy numbers of PPIA as control gene.

### Western Blot Analysis

Sodium dodecyl sulfate-polyacrylamide gel electrophoresis (SDS-PAGE) and immunoblotting were performed according to standard procedures. Briefly, tissue samples were lysed in RIPA buffer. Equal amounts of proteins (40 μg/lane), as determined by Lowry assay (Bio-Rad), were resolved by SDS-PAGE (7.5% acrylamide for ZO-1 and MLCK; 12.5% acrylamide for pMLC; 14% acrylamide for claudin-5). Membranes were incubated with antibodies specific to claudin-5 (1:500, 35–2500, Invitrogen), ZO-1 (1:500, 10,342,463, Fisher Scientific), MLCK (1:3000, M7905, Sigma), phospho-MLC (1:400, #3671s, cell signaling technologies) and GAPDH (1:10,000, ACR001PT, Acris Antibodies), respectively, and appropriate species-specific secondary infrared dye-conjugated antibodies (1:15,000; Li-cor) to reveal protein band densities using the Odyssey SA Imaging System and quantification with Image Studio (both Li-cor).

### Statistical Analysis

Statistical analysis was performed using Sigma Plot 13 software package (Systat Software, USA). To determine the required sample size, an a priori power analysis using G*Power [15] was performed using data from previously published studies. The a priori power analysis for the effect size of 0.7 suggests that a standard statistical power (1-β) of *P* = 0.95 for a given significance level (*α*) of 0.05 and four experimental groups can be obtained for brain damage with *n* = 11, brain water content with *n* = 10, and BBB integrity with *n* = 7 subjects per group. Exact Wilcoxon-Mann-Whitney tests were used for comparisons. MLCK western blotting data was tested for outliers by Rout outlier test (Q 1%). Based on the results, one data point was removed from the ML-7 data set (Fig. 4). For each outcome, *p* values were adjusted for multiple testing using Bonferroni-Holm correction. Results are presented as mean ± standard deviation (S.D.).

## Results

### Influence of SAH and ML-7 Application on Physiological Parameters

**Fig. 1:**
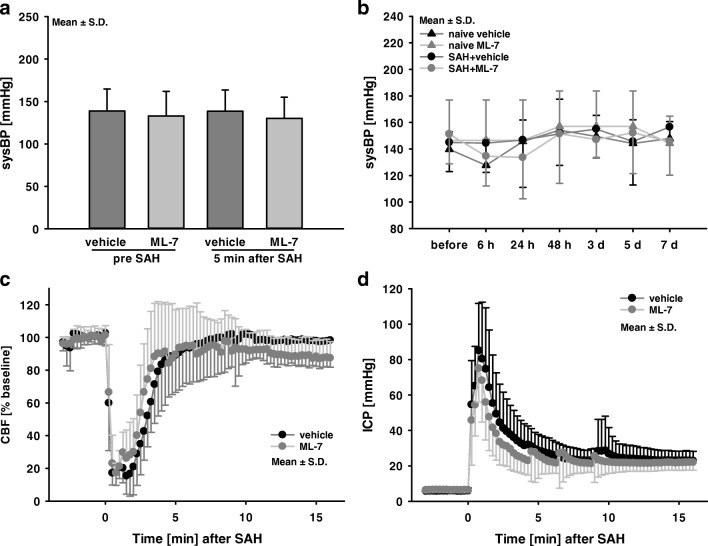
Systolic blood pressure, regional cerebral blood flow and intracranial pressure. Non-invasively determined systolic blood pressure (sysBP) did not differ between experimental groups during surgery (**a**) and in the seven-day observation period (**b**). SAH resulted in a decrease of cerebral blood flow (CBF, **c**) and an increase of intracranial pressure (ICP, **d**). Within the next 15 min, CBF recovered to 90% of baseline (**c**) and ICP remained elevated at 25 mmHg (**d**). Data are presented as mean ± S.D. and were not different between experimental groups

Physiological parameters (pH, blood glucose level, hemoglobin, hematocrit, and electrolyte levels) 24 h after SAH were within normal range and showed no differences between experimental groups (Table 1). Body temperature and systolic blood pressure (Fig. 1a) during surgery were within physiologic limits and stable over the 7-day observation period in all groups (Fig. 1b). ML-7 administration had no influence on blood pressure, body weight, or physiological parameters (pH, blood glucose level, hemoglobin, hematocrit, and electrolytes).

**Table 1:** Physiological parameters

	Vehicle+SAH	ML-7+SAH	Vehicle native	ML-7 native
pH	7.3 ± 0.1	7.4 ± 0.1	7.3 ± 0.1	7.3 ± 0.1
Hemoglobin (mg/dl)	13.2 ± 1.0	13.0 ± 0.8	12.7 ± 0.8	12.8 ± 0.6
HCT (%)	40.0 ± 3.1	40.0 ± 2.4	39.1 ± 2.4	39.4 ± 1.8
Na^+^ (mmol/dl)	133.3 ± 13.2	140.3 ± 11.0	131.0 ± 12.1	132 ± 18.1
Blood glucose levels (mg/dl)	404 ± 127	392 ± 35	273 ± 53.0	290 ± 18.2
Ca^2+^ (mmol/dl)	1.3 ± 0.1	1.4 ± 0.1	1.2 ± 0	0.8 ± 0
sysRR (mmHg) before SAH	139 ± 26	133 ± 29		
sysRR (mmHg) after SAH	134 ± 29	128 ± 27		
Rect. temp. (°C) during SAH	37.2 ± 0.3	37.4 ± 0.3		

*sysRR* non-invasively determined systolic blood pressure, *Rect. temp* rectal temperature

### Regional Cerebral Blood Flow and Intracranial Pressure

Successful SAH induction was confirmed by a rapid increase in ICP and decrease of CBF. Baseline ipsilateral regional CBF values were obtained 10 min before the induction of SAH. In all animals, SAH resulted in a CBF decrease to 20–30% of baseline followed by gradual recovery over 15 min to 90% of baseline. CBF data during surgery did not differ between vehicle and ML-7-treated mice (Fig. 1c).

After vessel perforation, ICP values increased in all animals to approximately 85 mmHg and remained elevated at 25 mmHg for 15 min (Fig. 1d). ICP data during surgery and until 15 min did not differ between the SAH groups. After 24 h, ICP values of ML-7-treated animals were significantly lower compared to vehicle (SAH+vehicle 12.0 ± 3.6 mmHg; SAH+ML-7 5.7 ± 2.3 mmHg, Fig. 3d).

### Influence of SAH on Ipsilateral Hippocampus mRNA Expression

**Fig. 2:**
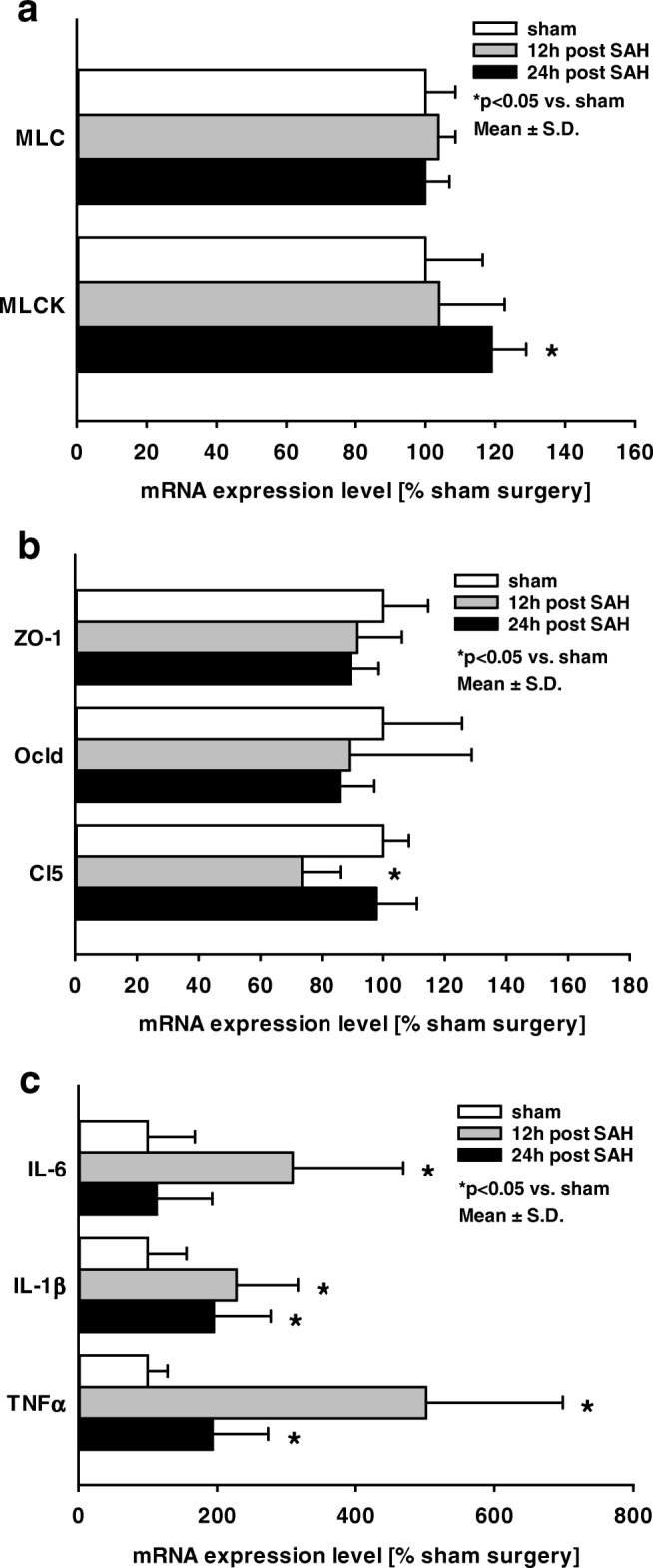
Influence of SAH on MLC and MLCK, tight junction protein, and inflammation marker gene expression. To determine the impact of murine SAH on mRNA expression of key proteins, qPCR was performed in samples from hippocampus at 12 and 24 h after SAH and compared to sham. In ipsilateral hippocampus, mRNA expression of MLCK increased significantly compared to sham (**a**). The analysis of tight junction protein mRNA expression showed that the expression of claudin5 (Cl5) was decreased 12 h after SAH (**b**). As markers for inflammation, TNFα, IL-6, and IL-1β were quantified. Expression of all genes peaked at 12 h after SAH in comparison to sham-operated animals (**c**). Twenty-four hours after SAH, mRNA expression of IL-6 returned to sham level, whereas IL-1β expression remained un-changed compared to 12 h post-insult. The expression of TNFα was lower compared to 12 h, but significantly compared to sham. Data are presented as mean ± S.D.

To determine the impact on the mRNA expression of key proteins, qPCR was performed in hippocampal samples at 12 and 24 h after SAH and compared to the sham group (Fig. 2). In the ipsilateral hippocampus, mRNA expression of MLCK increased significantly compared to sham (24 h SAH 119 ± 10%, *p* < 0.05; Fig. 2a). Analysis of tight junction protein mRNA expression showed that claudin 5 (Cl5) expression was decreased 12 h after SAH (12 h SAH 74 ± 19%; Sham 100 ± 24%; *p* < 0.05 vs. sham; Fig. 2b). As markers for inflammation, TNFα, IL-6, and IL-1β expression was quantified. The expression of all genes was significantly increased at 12 h after SAH in comparison to the sham group (TNFα 501 ± 197%; IL-6 309 ± 159%; IL-1β 228 ± 89%; *p* < 0.05 vs. sham; Fig. 2c). Twenty-four hours after SAH, mRNA expression of IL-6 returned to sham level, whereas IL-1β expression remained unchanged compared to 12 h post-SAH (195 ± 82%; *p* < 0.05 vs. sham). TNFα mRNA expression was lower compared to 12 h but still significantly higher compared to sham values (194 ± 80%).

### Effect of MLCK Inhibition on Brain Water Content 24 h after SAH

**Fig. 3:**
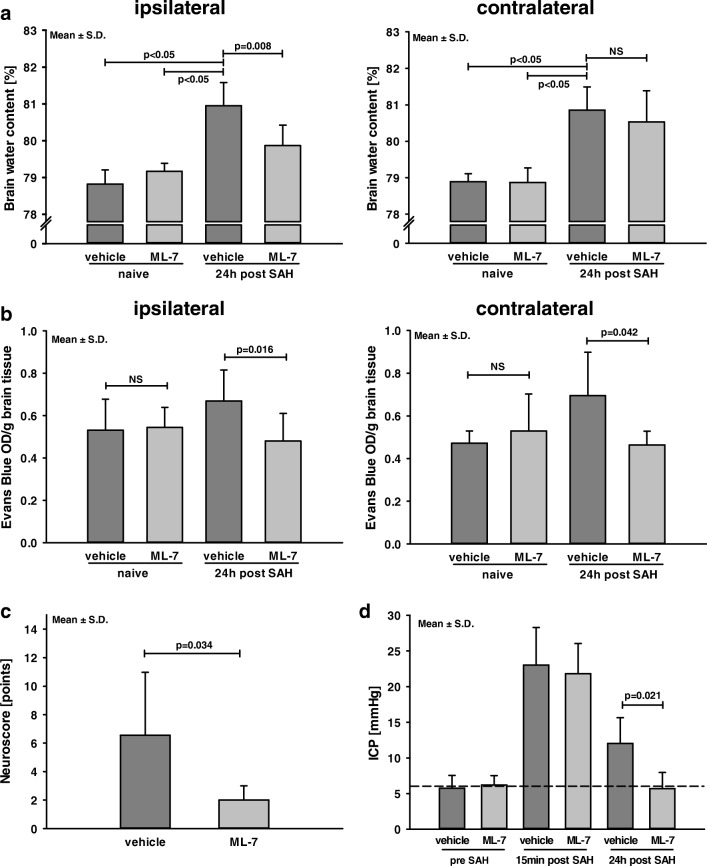
Influence of MLCK inhibition on brain edema formation, BBB integrity, functional outcome, and intracranial pressure. (**a**) Influence of MLCK inhibition by selective blockage with ML-7 on brain edema formation was examined by wet-dry ratio 24 h after SAH. Following SAH brain water content increased in vehicle treated animals. Twenty-four hours after SAH, brain water content was lower in ML-7-treated animals. (**b**) BBB functionality was assessed by Evans blue extravasation 24 h after SAH. Inhibition of MLCK resulted in less intracerebral Evans blue compared to vehicle-treated animals. (**c**) Neurological function was determined with a 31-point neuroscore and shows a less functional impairment in ML-7-treated animals. (**d**) Twenty-four hours after SAH ICP of ML-7-treated animals were lower compared to the vehicle group. Data are presented as mean ± S.D. *NS* not significant

Brain water content was determined as the wet/dry ratio in naive mice and in SAH mice 24 h after surgery. In non-operated animals, water content of both hemispheres was not different between vehicle or ML-7 group (ipsilateral: naive+vehicle 78.8 ± 0.3%; naive+ML-7 79.2 ± 0.2%, Fig. 3a; contralateral: naive+vehicle 79.0 ± 0.2%; naive+ML-7 78.8 ± 1.1%). Twenty-four hours after SAH, brain water content increased significantly in both hemispheres (ipsilateral: SAH+vehicle 81.0 ± 0.6%; *p* = 0.021 vs. naive+vehicle; contralateral: SAH+vehicle 80.9 ± 0.6%; *p* = 0.021 vs. naive+vehicle; Fig. 3a). In the presence of ML-7, increase of brain water content was significantly less in the ipsilateral hemisphere (SAH+ML-7 79.9 ± 0.6%; *p* = 0.024 vs. SAH+vehicle; Fig. 3a). Water content of the contralateral hemisphere did not differ between treatment groups (SAH+ML-7 80.5 ± 0.9%; SAH+vehicle 80.9 ± 0.6%; Fig. 3a).

### Blood-Brain Barrier Integrity 24 h after SAH

To further evaluate blood-brain barrier (BBB) integrity, the permeability to Evans blue dye was quantified. Specifically, Evans blue dye was injected and quantified 24 h after SAH in both hemispheres. In non-operated animals, Evans Blue extravasation did not differ between ML-7 and the vehicle group (ipsilateral: naive+vehicle 0.53 ± 0.14 OD/g; naive+ML-7 0.54 ± 0.1 OD/g, Fig. 3b; contralateral: naive+vehicle 0.47 ± 0.06 OD/g; naive+ML-7 0.53 ± 0.17 OD/g). After SAH, inhibition of MLCK with ML-7 resulted in a significantly lower amount of Evans Blue in both hemispheres compared to vehicle-treated animals (ipsilateral: SAH+vehicle 0.67 ± 0.15 OD/g; SAH+ML-7 0.48 ± 0.13 OD/g, *p* = 0.016; contralateral: SAH+vehicle 0.69 ± 0.20 OD/g; SAH+ML-7 0.46+0.06 OD/g, *p* = 0.042, Fig. 3b).

### Influence of MLCK Inhibition on Functional Outcome and Intracranial Pressure 24 h after SAH

Neurological function was tested with a 31-point neuroscore before surgery and 24 h after SAH (0 points = no impairment and 31 points = maximum impairment). In all groups, mice showed impairment after SAH. Nevertheless, neurological outcome was significantly improved in the ML-7 group (SAH+vehicle 6.6 ± 4.4 points; SAH+ML-7 2.9 ± 1.7 points; *p* = 0.034; Fig. 3c).

In naïve animals and 15 min after induction of SAH, intracranial pressure (ICP) was not different between ML-7 and vehicle-treated animals. After 24 h, ICP values of ML-7-treated animals were significantly lower than the vehicle group (SAH+vehicle 12.0 ± 3.6 mmHg; SAH+ML-7 5.7 ± 2.3 mmHg, *p* = 0.021; Fig. 3d).

### Influence of MLCK Inhibition on Tight Junction Proteins, MLC, and MLCK at 24 h After SAH

**Fig. 4:**
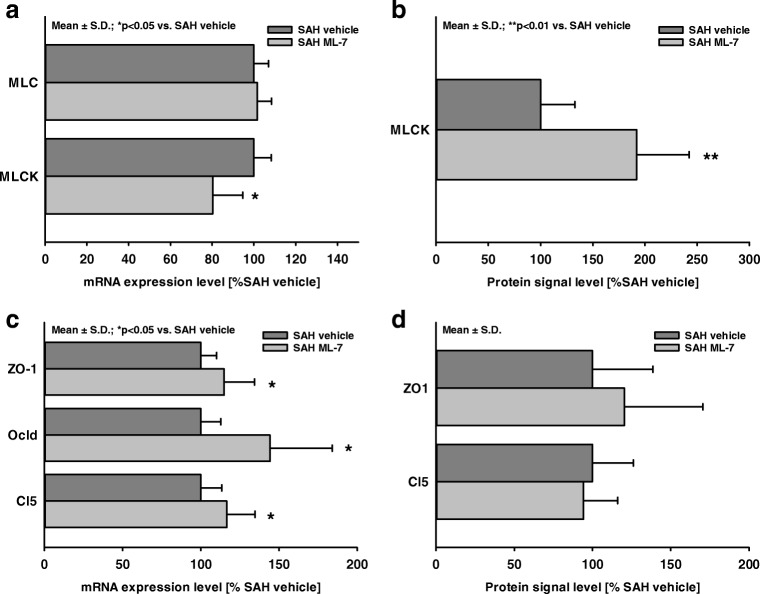
Influence of MLCK inhibition on MLC, MLCK, and tight junction mRNA and protein levels. Twenty-four hours after SAH, brain tissue was sampled from mice with ML-7 and vehicle treatment to determine mRNA and protein levels of MLC, MLCK, and tight junction proteins. (**a**) MLC mRNA levels were not influenced by ML-7, whereas MLCK mRNA expression decreased significantly. (**b**) In contrast to mRNA data, MLCK protein levels were twofold in ML-7-treated animals. (**c**) Tight junction proteins ZO-1, claudin 5 (Cl5), and occludin (Ocld) mRNA expression levels increased significantly in response to ML-7. (**c**) In contrast, ZO-1 and Cl5 protein levels were not significantly influenced by ML-7. Data are presented as mean ± S.D.

MLC and MLCK expression was determined 24 h after SAH. MLC mRNA level was not different between ML-7 and vehicle-treated animals, whereas MLCK mRNA expression was significantly lower (SAH+ML-7 80 ± 14%, *p* < 0.05 vs. vehicle; Fig. 4a**).** However, on the protein level, MLCK signal was significantly enhanced in ML-7-treated animals (Fig. 4b).

To identify the relationship between tight junction remodeling and BBB integrity, mRNA expression of the tight junction proteins zonula occludens-1 (ZO-1), claudin 5 (Cl5), and occludin (Ocld) was analyzed 24 h after SAH in animals treated with vehicle or ML-7. In the ipsilateral hippocampus ZO-1, Cl5 and Ocld expression increased in response to ML-7 treatment (SAH+ML-7: Cl5 117 ± 18%; Ocld 144 ± 40%; ZO-1 115 ± 19%, *p* < 0.05 vs. SAH vehicle; Fig. 4c). In contrast to mRNA expression, ZO-1 and Cl5 protein levels (Fig. 4d) were not different between ML-7- and vehicle-treated animals.

### Influence of MLCK Inhibition on Protein Expression in Brain Endothelial Cells

**Fig. 5:**
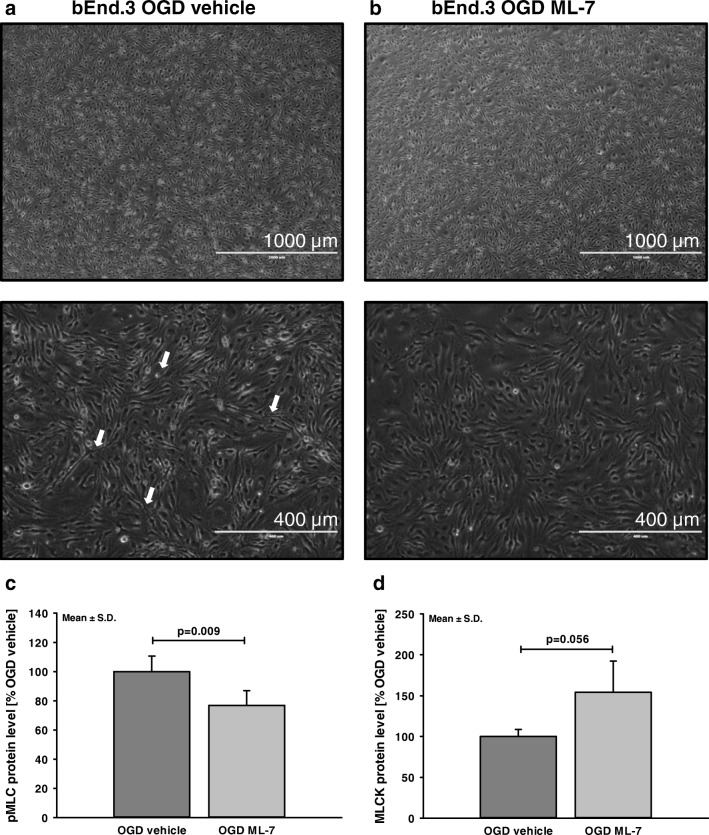
Influence of ML-7 on MLC phosphorylation and MLCK protein levels. To determine the influence of ML-7 in brain endothelial cells, bEnd.3 cells were subjected to 4 h OGD. (**a**) OGD resulted in pronounced cell shrinkage (white arrows). Pictures show endothelial cells at × 4 and × 10 magnification. (**b**) In the presence of ML-7, cells demonstrate less shrinkage pattern. (**c**) In ML-7-treated cells, phosphorylated MLC (pMLC) protein signal was significantly lower, whereas (**d**) MLCK protein level was by trend higher in ML-7-treated cells. Data are presented as mean ± S.D.

Unexpectedly, ML-7 increased MLCK protein levels after SAH. To rule out that this regulation in turn alleviates the effect of ML-7 on MLCK, we subjected brain endothelial cells (bEnd.3 cells) to 4 h of OGD in the presence of either the MLCK inhibitor ML-7 or vehicle solution. Four hours of OGD resulted in visible shrinkage of the cells (Fig. 5a), which appeared to be less pronounced in bEnd.3 cells treated with ML-7 (Fig. 5b) suggesting less contraction and activation of the contractile apparatus. Next, we determined the key target proteins of ML-7, pMLC, and MLCK. The phosphorylated MLC (pMLC) protein signal was significantly decreased in ML-7-treated cells (OGD+ML-7 77 ± 10% vs. vehicle; *p* < 0.01; Fig. 5c). Similar to ML-7-treated animals, MLCK protein levels was (by trend) higher in ML-7-treated cells (OGD+ML-7 154 ± 38%; *p* = 0.056 vs. vehicle; Fig. 5d). This MLCK regulation did not influence the effect of ML-7 on pMLC levels and cell shape in bEnd.3 cells subjected to OGD.

### Influence of MLCK Inhibition on Functional Outcome and Mortality over 7 Days Following SAH

**Fig. 6:**
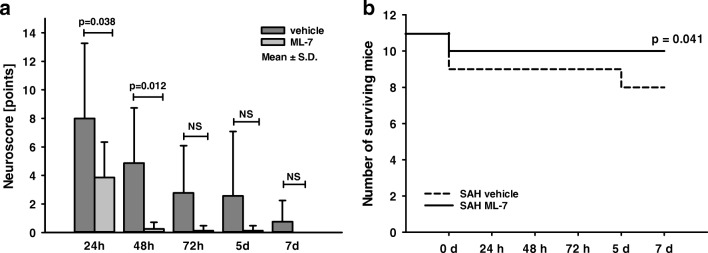
Influence of ML-7 on mortality rate and neurological recovery after SAH. Mice were subjected to SAH and treated with ML-7 or vehicle. (**a**) Neurological function was quantified with a 31-point scale during the 7-day observation period. ML-7-treated animals exhibited less neurological deficits on days 1 and 2 compared with vehicle. (**b**) Mortality rate (Kaplan-Meier curve) was significantly lower in ML-7-treated animals after SAH. Data are presented as mean ± S.D. *NS* not significant

Neurological function was tested with a 31-point neuroscore before and at day 1, 2, 3, 5, and 7 following SAH. In all experimental groups, animals showed impairment after SAH, but recovered within 72 h. The administration of ML-7 significantly improved neurological function 24 and 48 h after SAH (24 h: SAH+vehicle 7.3 ± 5.3 points; SAH+ML-7 5.8 ± 4.4 points, *p* = 0.038; 48 h: SAH+vehicle 4.3 ± 4.0 points; SAH+ML-7 1.1 ± 1.9 points, *p* = 0.012; Fig. 6a). Independent of the treatment arm, animals lost 13% of body weight within 24 h after SAH. Weight loss reached a maximum on the third day (− 19%). Thereafter, mice regained body weight to values prior to SAH. No differences were present between groups (data not shown).

The mortality rate of the vehicle group was 27% (3/11) 7 days post-SAH, in agreement with our previously published results in C57BL6 mice [9]. ML-7 significantly reduced mortality rate to 9% (1/11; *p* = 0.041, Fig. 6b).

### Influence of MLCK Inhibition on Neuronal Cell Count 7 Days after SAH

We analyzed the influence of reduced brain edema and intracranial pressure on neuronal brain damage. Neuronal cell count in hippocampus (CA1–3) at day 7 after SAH was not different between the experimental groups (data not shown). Only one mouse in the vehicle group exhibited pronounced neuronal death in the hippocampal regions CA1–3 and cortical brain tissue, suggesting a focal damage produced by the ICP sensor.

## Discussion

Brain edema formation plays an important role in the pathophysiology of ischemia-induced brain injury. However, the mechanisms by which vascular cells contribute to the breakdown of the blood-brain barrier (BBB) in SAH remain elusive. We demonstrate for the first time that blocking the intracellular contraction apparatus with a myosin light chain kinase inhibitor (1) preserves blood-brain barrier integrity, (2) reduces brain edema formation, (3) decreases elevated intracranial pressure, (4) improves neurological function, and (5) reduces mortality following SAH.

The murine endovascular perforation model used in the current study replicates the key features of human SAH. The model causes delayed cerebral vasospasm, neurological dysfunction, brain edema, and a mortality of ∼ 30% [9, 16–18]. Cerebral brain edema is considered the cardinal pathophysiological event underlying cell death and severe complications after SAH [1]. The initial rise of ICP and decrease of CBF are initiating factors for the development of cytotoxic edema. Persistent ischemia gives rise to endothelial apoptosis and breakdown of the BBB. Disruption of the BBB results in vasogenic edema, which leads to further increase of ICP and reduction of CBF [3]. The mechanisms of BBB disruption after SAH, especially the role of the endothelial barrier, are still unclear.

The BBB comprises endothelial cells, pericytes, and astrocytes. Cerebral endothelial cells are joined by tight junctions, which ensure the integrity of the BBB and limit paracellular influx of hydrophilic molecules across the BBB. SAH increases the permeability of the BBB, which permits extravasation of water into the brain [19]. Increased permeability could be a result of the activation of the contractile apparatus. The contractile apparatus of, e.g., endothelial cells is directly connected to tight junction proteins. Therefore, the integrity of the BBB may be influenced by an upregulation of MLCK, which leads to increased phosphorylation of MLC. In turn, MLC phosphorylation facilitates cytoskeletal rearrangement, which may reduce endothelial cell-to-cell contact [20]. The hypothesis is supported by previous findings that hypoxic-dependent activation of the MLCK leads to increased permeability of endothelial cells and to increased MLC phosphorylation. Importantly, administration of the MLCK inhibitor ML-7 counteracts both adverse events. In addition, ML-7 significantly reduces brain water content in a two-vein occlusion stroke model and after traumatic brain injury [4, 7]. These results are consistent with the present study where inhibition of MLCK stabilized BBB and prevented brain edema formation 24 h after SAH. Reduced brain edema was accompanied by a significant reduction in ICP that improved outcome and reduced mortality.

Our data indicate that mRNA expression of MLCK is increased after SAH compared to sham surgery and that selective MLCK inhibition after SAH improves outcome of SAH. Other groups have not reported a difference in the myosin light chain kinase activity of canine basilar artery between a control study and two models of SAH for up to 10 days after intracisternal injection of blood [21]. Whereas there is no increased activity of MLCK in the basilar artery, the regulatory pathway of Ca^2+^-calmodulin-MLCK is the major candidate for arterial vasospasm after SAH. Phosphorylation of MLC is increased during vasospasm after SAH in the anterior spinal artery [22] and basilar artery [23]. In addition, vasospasm is reversed by the topical application of MLCK inhibitor ML-9 [24]. To demonstrate the influence of ML-7 on endothelial cells, we performed in vitro OGD experiments. In these cells, post-hypoxic shrinkage of the cellular shape was reduced by ML-7. In line with the visual changes in cell form, we show that inhibition of MLCK reduced the phosphorylation of MLC. The data support the assumption that post-ischemic activation of MLCK results in cytoskeletal rearrangement and reduced endothelial cell-to-cell contact and tightness of the blood-brain barrier integrity.

In addition, we investigated the morphological features of brain damage. We hypothesized that pronounced reduction in ICP combined with brain edema should result in not only functional impairment but also histological evidence of injury. Unfortunately, only one mouse in the vehicle group exhibited histopathological signs of substantial neuronal death. The shape of the morphological brain damage suggests that the damage was caused by the ICP sensor. At 7 days post-SAH, there was no reduction in the neuronal cell count in the hippocampus. We cannot rule out, however, that damaged neurons were not sampled or visualized using standard light microscopic methods. However, similar results were reported by other groups using experimental endovascular models in, e.g., rats [25]. Although the present model failed to produce sustained substantial neuronal cell loss, other features of SAH such as increase in ICP, drop in CBF, neurological deficits, and pronounced brain edema were reliably reproduced.

The present study may be limited by the fact that integrity of the BBB was only investigated within 24 h after SAH. In the literature, increased brain water content was found in the cortex 24 to 48 h after SAH with a peak at 24 h [26, 27]. Microvascular basal lamina damage was demonstrated from 24 to 72 h after SAH [26]. In the present study, we aimed to investigate brain edema at its peak and, therefore, at 24 h. Furthermore, no time points later than 7 days after SAH were investigated, because neurological function and histopathological damage did not reveal significant differences 7 days after SAH.

Unexpectedly, MLCK protein levels were increased by ML-7 in animals and by trend in the in vitro experiments. In the endothelial cells, we were able to demonstrate that higher MLCK levels did not have an effect on pMLC levels and that ML-7 retained its effect on MLCK and pMLC. We therefore postulate that the MLCK upregulation is a compensatory regulation, which may represent regulation on protein level to reestablish higher pMLC levels. The influence of ML-7 on pMLC was determined in brain endothelial cells in vitro. We therefore cannot rule out that the in vivo regulation may be different.

In addition, MLCK is not only expressed in endothelial cells but also in various cell types (for review please see [28]) in the brain. It is also found in astrocytes [29], neurons [30], and microglia [31]. ML-7 targets MLCK in all cells and has even effects on Ca^2+^ currents in pericytes [32]. The effect of ML-7 is therefore not limited to endothelial cells. However, the endothelial layer is the most important barrier to seal the brain from the circulation.

In summary, the present study indicates that the endothelial cell cytoskeleton plays a critical role in barrier function and integrity of the BBB. By selectively inhibiting MLCK with ML-7, we have demonstrated that the endothelial cytoskeleton contributes to increased permeability of the BBB after SAH and vasogenic edema formation. The present data therefore provide convincing evidence of the interplay between endothelial dysfunction with tightness of the neurovascular unit. Accordingly, MLCK may serve as a promising target for the treatment of vasogenic brain edema after SAH.

## Electronic Supplementary Material

ESM 1 (DOCX 15 kb)
