# Supplementary material for: The Contractile Apparatus Is Essential for the Integrity of the Blood-Brain Barrier After Experimental Subarachnoid Hemorrhage
Source: Transl Stroke Res. 2018 Nov 23;10(5):534–45. doi: 10.1007/s12975-018-0677-0 (PMC6733822; doi:10.1007/s12975-018-0677-0)
Supplement: Supplementary file 1 — (DOCX 15 kb) [file 12975_2018_677_MOESM1_ESM.docx]

**Supplemental DATA**

| **Supplemental Table 1.** Criteria of the neuroscore – 31 points score. | |
| --- | --- |
| **1. General behavioral deficit**  Consciousness explores spontaneously (0)  awake, but passive (1)  no attempts (comatose) (2)  **2. Cranial nerve reflexes**  Whisker (movement) present (0)  absent (1)  Hearing (reacts to hand clapping)  present (0)  absent (1)  **3. Motor deficit**  Front leg left normal (0)  stiff (1)  paralyzed (2)  Front leg right normal (0)  stiff (1)  paralyzed (2)  Rear leg left normal (0)  stiff (1)  paralyzed (2)  Rear leg right normal (0)  stiff (1)  paralyzed (2) | **4. Coordination**  Placing test^a^ present (0)  absent (5)  Beam walking (3 cm) Score (0-4)  Beam walking (1.5 cm) Score (0-4)  Beam walking (1 cm) Score (0-4)  Criteria:  0 points: normal movement /no impairment  1 point: feet misplacements  2 points: sits down/ stops moving  3 points: falls after a few steps  4 points: falls immediately  **5. Neuroscore (Bederson)**  No impairment (0)  Bending of the front paw (1)  Reduced resistance from lateral (2)  Circling pulling at the tail (3)  Spontaneous circling (4)  No spontaneous movement (5) |
| This score comprises broad criteria that assess for general functional impairment. Mice are rated according to the listed items. Points are assigned for impaired function. The scores of each subitem are added to a total sum. High scores indicate an impairment of neurological function (total number of points: 31).  ^a^ front paws reach towards the ground when lifted by the tail. | |
